# A new record of the genus *Froggattiella* Leonardi (Hemiptera, Coccomorpha, Diaspididae) in South Korea

**DOI:** 10.3897/BDJ.11.e110948

**Published:** 2023-10-10

**Authors:** Hyeong Su Kim, Jaeyun Kim, Wonhoon Lee

**Affiliations:** 1 Department of Plant Medicine and Institute of Agriculture and Life Sciences, Gyeongsang National University, Jinju, South Korea Department of Plant Medicine and Institute of Agriculture and Life Sciences, Gyeongsang National University Jinju South Korea; 2 Institute of Agriculture and Life Science, Gyeongsang National University, Jinju, South Korea Institute of Agriculture and Life Science, Gyeongsang National University Jinju South Korea

**Keywords:** Odonaspidini, New mention, Asia, Armored scale

## Abstract

**Background:**

The genus *Froggattiella* Leonardi, 1900 belongs to the family Diaspididae, and five species of *Froggattiella* have been recorded worldwide. In this study, *Froggattiellapenicillata* (Green, 1905), which attacks bamboos, is newly recorded in South Korea. The colonies of *F.penicillata* were collected on a bamboo forest located in Gajwa-dong, Jinju-si, Gyeongsangnam-do, South Korea (35.1599, 128.1029). Description of the adult female, host plant, adult female illustrations, and global distribution of this species are provided.

**New information:**

*Froggattiellapenicillata* (Green, 1905) is reported for the first time in South Korea. This species occurrs under sheathing bases of the leaves and is observed attached on the stem and not on the leaf.

## Introduction

The family Diaspididae (Hemiptera, Coccoidea) is the most diverse family in the Coccoidea, with more than 2,700 species of 418 genera worldwide (García Morales et al. 2016). The genus *Froggattiella* Leonardi, 1900 belongs to the subfamily Aspidiotinae and the tribe Odonaspidini (Normark et al. 2019) and is a small group including five species in the world: *F.gigantochloae* Aono 2009, *F.pentapeniculata* Aono, 2009, *F.inusitata* (Green, 1896), *F.mcclurei* Ben-Dov, 1988, and *F.penicillata* (Green, 1905) (Green 1905, Ben-Dov 1988, Aono 2009). These species have been recorded from mostly zoogeographical regions (Ben-Dov 2015, García Morales et al. 2016). There are only found on Poaceae, but there are mainly bamboo-feeding species (Amouroux et al. 2020).

*Froggattiellapenicillata* (Green, 1905) was originally described from bamboo, namely *Gigantochloaaspera* (Schult. & Schult.f.) Kurz in Paradeniya, Sri Lanka (Ben-Dov 1988), since then, it has been recorded only from bamboo in various territories of Asian, African, and American countries (Ben-Dov 1988). This species was intercepted most frequently in quarantine at U.S. ports of entry on bamboo from the Caribbean islands, China, Japan, South and Central America, and Vietnam (Miller and Davidson 2005). In South Korea, it was detected in the quarantine inspection of bamboo imported from China at the port of entry (Suh and Gregory 2007, Suh 2016). To date, information related to the life cycle and damage of this species has not been well known.

In the present paper, we report *F.penicillata* for the first time in South Korea and provide an identification key to adult females of seven armored scales in Korea that are found on bamboos.

## Materials and methods

These specimens were collected in Mt. Gajwa, Jinju-si, Gyeongsangnam-do, South Korea (35.1599, 128.1029), and were identified on collecting the part from the stem of the host plant (Poaceae: *Sasaquelpaertensis* Nakai). The samples of adult females were preserved in 90% ethanol. The specimens were examined under an optical microscope (DM6, LEICA, Germany) and habitus photographs were taken using a stereoscopic microscope (M205C, LEICA, Germany). To prepare slide-mounted specimens, samples were placed in hot 10% potassium-hydroxide (KOH) solution for 30 minutes or 1 hour at 70°C. After then, samples were put in distilled water for 5 minutes, and few drops of stain was added to distilled water and stayed for 5 minutes (Miller and Davidson 2005). The identification of adult females was conducted, based on Ben-Dov (1988). Permanent slide mounts of adult females (ID: 230131HS148) were deposited in the Institute of Agriculture & Life Science, Gyeongsang National University, South Korea.

## Taxon treatments

### Froggattiella penicillata

(Green, 1905)

1FEBCECA-5687-5784-95C0-4487028B4392

*Odonaspispenieillata* Green, 1905: 346 - Green 1905
*Anoplaspispenicillata* (Green) Kuwana, 1933: 38 - Kuwana 1933
*Froggattiellapenicillata* (Green) Rutherford, 1915: 104 - Rutherford 1915

#### Materials

- **Type status:** Other material. **Occurrence:** recordedBy: Hyeongsu Kim; individualCount: 5; sex: female; lifeStage: adult; occurrenceID: 8AC36059-D135-5459-8C0B-16037B401B65; **Taxon:** scientificName: *Froggattiellapenicillata*; kingdom: Animal; phylum: Arthropoda; class: Insecta; order: Hemiptera; family: Diaspididae; genus: Froggattiella; specificEpithet: penicillata; **Location:** country: South Korea; stateProvince: Gyeongsangnam-do; municipality: Jinju-si; locality: Gajwa-dong 803; verbatimLatitude: 35°09'35.6"N; verbatimLongitude: 128°06'10.4"E; georeferenceProtocol: label; **Identification:** identifiedBy: Hyeongsu Kim; dateIdentified: 2023; **Event:** eventDate: 01/31/2023; **Record Level:** language: en; collectionCode: Insects; basisOfRecord: PreservedSpecimen
- **Type status:** Other material. **Occurrence:** recordedBy: Hyeongsu Kim; individualCount: 5; sex: female; lifeStage: adult; occurrenceID: 6D3CF3D9-AD8E-582B-8347-49CC64B12140; **Taxon:** scientificName: *Froggattiellapenicillata*; kingdom: Animal; phylum: Arthropoda; class: Insecta; order: Hemiptera; family: Diaspididae; genus: Froggattiella; specificEpithet: penicillata; **Location:** country: South Korea; stateProvince: Gyeongsangnam-do; municipality: Jinju-si; locality: Gajwa-dong 803; verbatimLatitude: 35°09'35.6"N; verbatimLongitude: 128°06'10.4"E; georeferenceProtocol: label; **Identification:** identifiedBy: Hyeongsu Kim; dateIdentified: 2023; **Event:** eventDate: 01/31/2023; **Record Level:** language: en; collectionCode: Insects; basisOfRecord: PreservedSpecimen
- **Type status:** Other material. **Occurrence:** recordedBy: Hyeongsu Kim; individualCount: 5; sex: female; lifeStage: adult; occurrenceID: FC5257E0-7B25-5DDA-AAB5-AC62229B3D91; **Taxon:** scientificName: *Froggattiellapenicillata*; kingdom: Animal; phylum: Arthropoda; class: Insecta; order: Hemiptera; family: Diaspididae; genus: Froggattiella; specificEpithet: penicillata; **Location:** country: South Korea; stateProvince: Gyeongsangnam-do; municipality: Jinju-si; locality: Gajwa-dong 803; verbatimLatitude: 35°09'35.6"N; verbatimLongitude: 128°06'10.4"E; georeferenceProtocol: label; **Identification:** identifiedBy: Hyeongsu Kim; dateIdentified: 2023; **Event:** eventDate: 01/31/2023; **Record Level:** language: en; collectionCode: Insects; basisOfRecord: PreservedSpecimen
- **Type status:** Other material. **Occurrence:** recordedBy: Hyeongsu Kim; individualCount: 5; sex: female; lifeStage: adult; occurrenceID: 8AC36059-D135-5459-8C0B-16037B401B65; **Taxon:** scientificName: *Froggattiellapenicillata*; kingdom: Animal; phylum: Arthropoda; class: Insecta; order: Hemiptera; family: Diaspididae; genus: Froggattiella; specificEpithet: penicillata; **Location:** country: South Korea; stateProvince: Gyeongsangnam-do; municipality: Jinju-si; locality: Gajwa-dong 803; verbatimLatitude: 35°09'35.6"N; verbatimLongitude: 128°06'10.4"E; georeferenceProtocol: label; **Identification:** identifiedBy: Hyeongsu Kim; dateIdentified: 2023; **Event:** eventDate: 01/31/2023; **Record Level:** language: en; collectionCode: Insects; basisOfRecord: PreservedSpecimen
- **Type status:** Other material. **Occurrence:** recordedBy: Hyeongsu Kim; individualCount: 5; sex: female; lifeStage: adult; occurrenceID: D55BE239-421C-55A9-A5D9-1959E4E36B6B; **Taxon:** scientificName: *Froggattiellapenicillata*; kingdom: Animal; phylum: Arthropoda; class: Insecta; order: Hemiptera; family: Diaspididae; genus: Froggattiella; specificEpithet: penicillata; **Location:** country: South Korea; stateProvince: Gyeongsangnam-do; municipality: Jinju-si; locality: Gajwa-dong 803; verbatimLatitude: 35°09'35.6"N; verbatimLongitude: 128°06'10.4"E; georeferenceProtocol: label; **Identification:** identifiedBy: Hyeongsu Kim; dateIdentified: 2023; **Event:** eventDate: 01/31/2023; **Record Level:** language: en; collectionCode: Insects; basisOfRecord: PreservedSpecimen

#### Description

**Figure 1.**
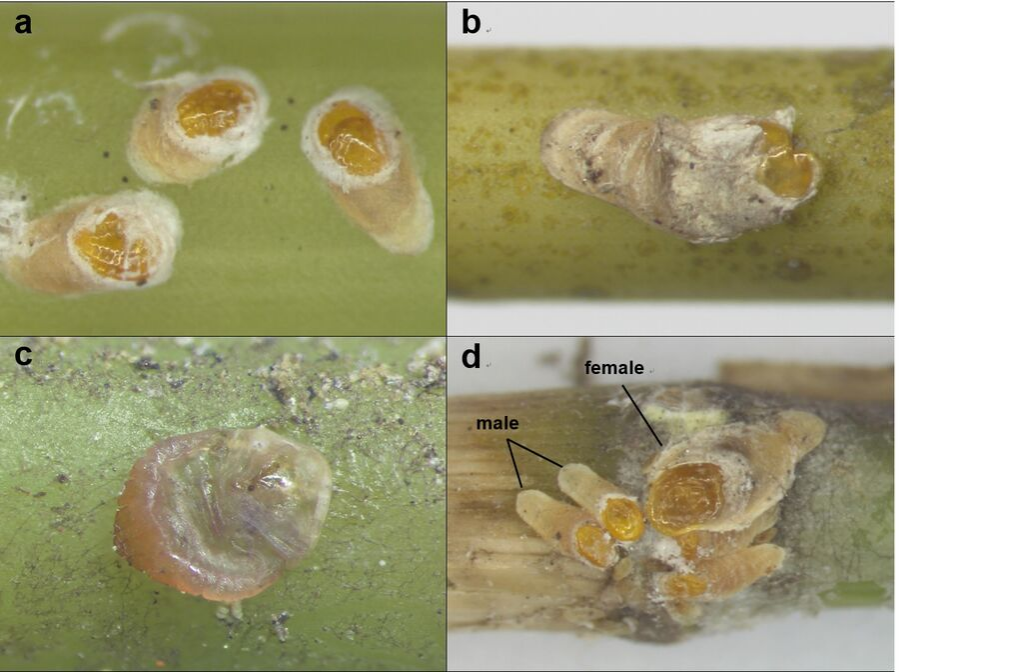
The habit of *Froggattiellapenicillata*. **a** second-instar female; **b** adult female; **c** Body of adult female; **d** adult female and male scale cover on plant.

**Figure 2.**
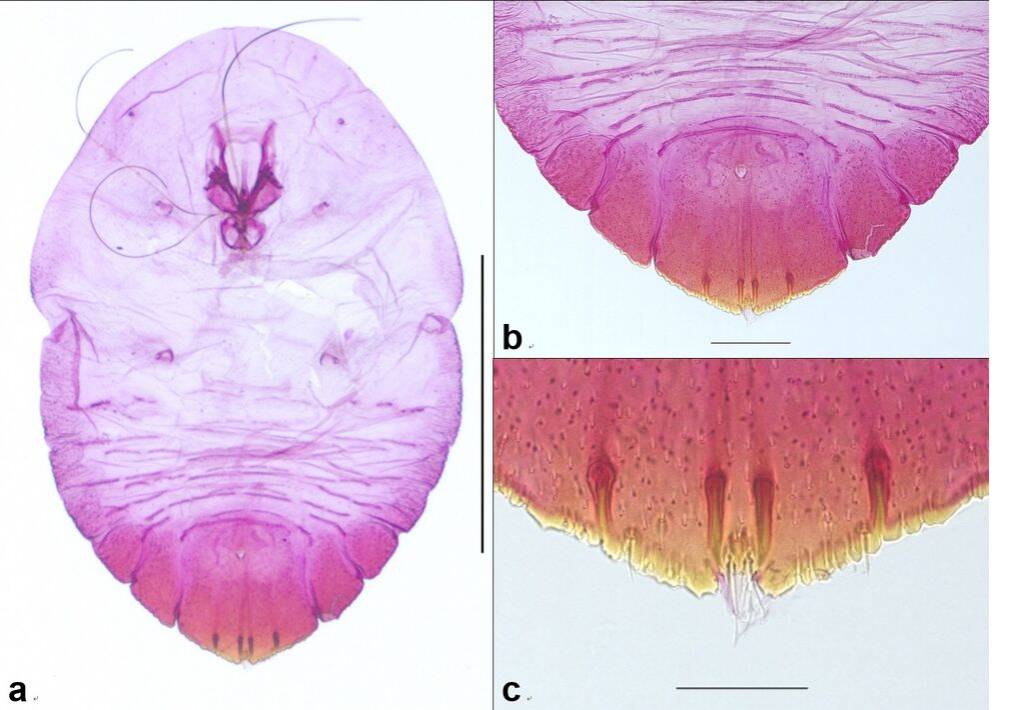
*Froggattiellapenicillata*. **a** adult female; **b** abdomen; **c** median lobe. Scale bars: a = 200 µm, b = 100 µm, c = 50 µm.

The scale covers of female adults are generally yellow in color, and the color of the larval exuviae, located at the front of the scale cover, is a deep yellow (Fig. 1); shed scale covering marginal; light purple or light brown. Adult male scale cover is smaller and narrower than that of female scale cover. The male cover looks more oval compared to that of the female cover. Color of the male cover is similar to the female cover. On the slide-mounted female, the body shape is round to oval (Fig. 2); Body length 1.07-1.18 (median 1.16) mm, Width 0.72-0.82 (median 0.8) mm; the end of the median lobes is pointed and there are approximately five to six gland spines between median lobes; ventral marginal setae slightly shorter than dorsal marginal setae in abdominal segment 8. Margin of the pygidium, with a pair of pygidial sclerosis. Perivulvar pores absent; marginal setae located on segment 4, 5, 6, and 7; Several creulae between dorsal abdominal segments. Anterior spiracle with approximately four or six spiracular pores, posterior spiracle without pores; many secretory ducts are concentrated on the dorsal and dorsal surfaces.

#### Distribution

Asia: South Korea (new record), Japan, China, Georgia, India, Iran, Pakistan, Philippines, Sri Lanka, Taiwan. Africa: Algeria, South Africa. North America: United States, Hawaii, Puerto Rico, Mexico. South America: Guyana, Jamaica. Oceania: Australia, Fiji, Palau (García Morales et al. 2016).

#### Notes

This species had been not found in South Korea before.

#### Host plants

*Sasaquelpaertensis* Kakai (in this study), *Arundinaria* sp., *Arundodonax* L., *Bambusablumeana* Schult.f., *Bambusamerrilliana* (Elmer) Rojo & Roxas, *Bambusamultiplex* (Lour.) Raeusch. ex Schult. & Schult.f., *Bambusapervariabilis* McClure., *Bambusavulgaris* (L.), *Dendrocalamusasper* (Schultes f.), *Dendrocalamuslatiflorus* Munro, *Gigantochloalevis* (Blanco) Merr., *Ochlandratravancorica* (Bedd.), *Panicum* sp., *Phyllostachysaurea* Carrière ex. A., *Phyllostachysbambusoides* Siebold & Zucc., *Phyllostachysedulis* (Carrie) J.Houz., *Phyllostachysnigra* (Lodd.) Munro, *Pleioblastusargenteostriatus* (Regel) Nakai, *Pseudosasahindsii* (Munro) S.L.Chen & G.Y.Sheng ex T.G.Liang, *Sasa* sp., *Schizostachyumglaucifolium* (Rupr.) Munro, *Schizostachyumlumampao* (Blanco), and *Spartina* sp. (García Morales et al. 2016).

## Identification Keys

### Identification key to adult females of armored scale insects on bamboos in South Korea (modified from Suh and Hodges (2007))

**Table:** 

1	Without two-barred macroducts on margin of abdomen	2
–	With two-barred macroducts on margin of abdomen	3
2	With perivulvar pores on pygidium of abdomen	*Odonaspissecreta* (Cockerell)
–	Without perivulvar pores on pygidium of abdomen	*Froggattiellapenicillata* (Green)
3	Median lobes yoked	*Pinnaspisbuxi* (Bouché)
–	Median lobes not yoked	4
4	Median lobes very small and pointed, occurring on the lower part of the leaf	*Unachionaspistenuis* Maskell
–	Serrated plates present anterior to 2^nd^ lobes	5
5	With less than 10 perivulvar pores on pygidium, occurring on the base of the leaf	*Kuwanaspishikosani* Kuwana
–	With more than 20 perivulvar pores on pygidium, occurring on the base of the stem	6
6	Abdominal segment 1 with transverse row of macroducts	*Kuwanaspishowardi* (Cooley)
–	Abdominal segment 1 without transverse row of macroducts	*Kuwanaspispseudoleucaspis* (Kuwana)

## Discussion

Bamboo is an important resource with ecological and economic value due to its versatility as food, wood, landscape, conservation, and ornamental uses (Mera and Xu 2014). Currently, ca. 150 species of armored scale insects have been known to feed on bamboo in Asian region (Ülgentürk et al. 2014). In South Korea, six species were reported as armored scale insects feeding on bamboo, and one species was newly added from this study: *Odonaspissecrata* (Cockerell, 1896), *Pinnaspisbuxi* (Bouché, 1851), *Unachionaspistenuis* Maskell, 1897, *Kuwanaspishikosani* Kuwana, 1902, *Kuwanaspishowardi* (Cooley, 1898), *Kuwanaspispseudoleucaspis* (Kuwana, 1923), and *Froggattiellapenicillata* (Suh 2016).

In South Korea, *F.penicillata* was collected on *Sasaquelpaertensis*, and colonies of *F.penicillata* occurred beneath the base of the leaves. *Sasaquelpaertensis* is a new host plant and has been not reported in the previous studies (García Morales et al. 2016). The new host plant suggests that *F.penicillata* can survive in a variety of host plants and has a high possibility of spreading.

Until now, economic damage, distribution, and biology about the seven species have been not known in South Korea. As bamboo has unique ornamental and aesthetic values, the worldwide trade of bamboo has been continuously increased, and it can make opportunity of associated scale insects’ invasions. Thus, intensive surveys of scale insects feeding bamboo are necessary to manage scale insects and detect potential invasive scale insects in South Korea.

## Supplementary Material

XML Treatment for Froggattiella penicillata
